# Clinical Decision Support May Link Multiple Domains to Improve Patient Care: Viewpoint

**DOI:** 10.2196/20265

**Published:** 2020-10-16

**Authors:** David Kao, Cynthia Larson, Dana Fletcher, Kris Stegner

**Affiliations:** 1 Department of Cardiology University of Colorado School of Medicine Aurora, CO United States; 2 UCHealth Aurora, CO United States; 3 Evida Clinical Consulting, Inc Golden, CO United States; 4 G(x)P Advisors, Inc Thornton, CO United States

**Keywords:** clinical decision support, population medicine, evidence-based medicine, precision medicine, care management, electronic health records

## Abstract

Integrating clinical decision support (CDS) across the continuum of population-, encounter-, and precision-level care domains may improve hospital and clinic workflow efficiency. Due to the diversity and volume of electronic health record data, complexity of medical and operational knowledge, and specifics of target user workflows, the development and implementation of comprehensive CDS is challenging. Additionally, many providers have an incomplete understanding of the full capabilities of current CDS to potentially improve the quality and efficiency of care delivery. These varied requirements necessitate a multidisciplinary team approach to CDS development for successful integration. Here, we present a practical overview of current and evolving applications of CDS approaches in a large academic setting and discuss the successes and challenges. We demonstrate that implementing CDS tools in the context of linked population-, encounter-, and precision-level care provides an opportunity to integrate complex algorithms at each level into a unified mechanism to improve patient management.

## Introduction

The US health care sector has marched steadily toward adoption and standardization of health information technology systems over the past decade with much anticipation of their potential [1]. Clinicians face the increasing challenge of incorporating new guidelines into clinical practice as the volume and complexity of electronic health data continue to grow [2-7]. However, evidence of improvements in the quality, safety, and efficiency of patient care stemming from electronic medical records (EMRs) is mixed [3,5,8].

Clinical decision support (CDS) systems are broadly defined as information and tools used in patient care, such as reminders, alerts, and guidelines [9]. Automated CDS is developed to assist clinician decision-making and improve patient care by leveraging the breadth of electronic health data combined with up-to-date practice recommendations in the context of local workflow requirements. Ideally, CDS applications will seamlessly translate enhanced decision-making into action to optimize health care delivery [10]. For maximal impact, information and processes are delivered to providers on an ongoing basis; ideally, they are fully integrated with institutional workflows [11,12].

The increasing investment in the combination of traditional evidence-based and precision medicine also requires innovation in CDS approaches [13]. Incorporating ‘omic medicine and collectively characterizing and measuring molecular data from fields including molecular diagnostics, environmental exposures, and lifestyle behaviors will require considerable assistance from EMRs, CDS tools, and other expert systems given the scope and complexity of the data involved. Given that contemporary EMRs are not equipped to capture and access ‘omic “big data,” one important function of CDS will be to access and use data from multiple disparate sources to generate recommendations ranging from discrete decisions such as choosing a medication to long-term chronic disease prevision. Additionally, future CDS applications should ideally operate using consistent, portable CDS knowledge bases to facilitate shared implementation and querying strategies between institutions that leverage data along the spectrum from highly individualized ‘omics to population-level evidence-based medicine without requiring multiple, possibly inconsistent implementations at different institutions.

CDS is uniquely positioned to support population-, encounter-, and precision-level medicine as a continuum of care delivery through EMR and clinical informatics systems. Implementing CDS tools across the spectrum of these three clinical care domains may potentially improve efficiency and quality of care for patients. Medicare bundled payments and other pay-for-performance models incentivize efficient and consistent care transitions from the emergency department (ED) to inpatient settings to outpatient settings [14]. Therefore, new CDS solutions should reflect the reality of integrated care delivery. However, significant effort is required in planning and development to ensure that CDS applications align with end-user workflows to maximize efficiency and provider uptake. In addition, effective provider education and change management are critical for CDS implementation. Here, we summarize and provide examples of the implementation of CDS tools within each clinical care domain as well as across all three domains and the challenges that were encountered during this implementation at a large medical center.

## Overview of the Three CDS Health Care Domains

### Population-Level CDS

The goal of population health is to improve long-term outcomes of patient cohorts by means of preventive interventions, patient engagement, care coordination, and other activities outside clinical visits. Population-level CDS requires integration with very different workflows that are unique to a broad range of providers, such as care managers, social workers, or patient outreach staff, all independent of discrete, face-to-face encounters. Potential population health CDS applications may include identifying target patients (eg, patient registries) and monitoring of long-term treatment profiles (eg, guideline adherence dashboards), clinical outcomes (eg, urgent clinic visits or hospitalization), and care transitions (eg, post-hospitalization follow-up). By leveraging data as it enters the EMR irrespective of timing, CDS can support many providers in surveilling and delivering integrated patient- and disease-focused care to target patient populations. The range of population health CDS is currently limited in part by a lack of clear and consistent workflows. As these standards evolve, CDS will likely become critical for successful population health management given the challenges of the unpredictable timing of important clinical events, complexity of data, and novel interpretation methods that will be required.

### Encounter-Level CDS

Encounter-level CDS is the most common and familiar type of CDS, and the most evidence has been obtained to date regarding its efficacy. Encounter-level applications generally provide important information, give reminders, or suggest a course of care during a discrete clinical interaction such as a clinic visit, a hospitalization, an elective procedure, or even a telephone call. This model allows providers to take immediate action that affects patient care, thereby providing the right information to the right person at the right time through the EMR, which is the right channel to enact a recommendation. Encounter-level CDS applications are often high-value use cases because practice guidelines, performance metrics, and safety measures often have important implications for patient outcomes, reimbursement, or public reporting of performance; also, direct suggestions within a clinical workflow (eg, when an order is placed) can change a provider’s action in a timely manner. Examples of encounter-level CDS include alerts regarding drug-drug or drug-allergy interactions, risk-based vital sign monitoring recommendations, or reminders in computerized provider order entry systems to place orders for tests or medications [8,15,16]. In addition, encounter-level CDS systems are increasingly able to support complex, interactive applications to standardize care delivery for specific clinical scenarios in accordance with evidence-based recommendations.

### Precision-Level CDS

Precision-level CDS integrates complex, voluminous, and disparate data regarding a patient’s specific characteristics in multiple domains to provide clinical guidance [17]. Although the term “precision medicine” is often associated with genetic- or genomic-guided medical therapy, it applies to any individualized management strategy based on a patient’s unique combination of traits, such as demographics, clinical and family history, physical traits (eg, weight, blood tests), activity, mental health, socioeconomic status, and environment, among many others [18]. Telemonitoring and remote care delivery education are also considered to be precision medicine tools in chronic disease management that are intended to optimize access to care and empower patients to manage their own health [19,20]. These tools facilitate communication between the patient and the provider about the patient’s individualized risks and needs; they also formulate beneficial and achievable treatment plans and provide personalized education.

## Applications of CDS Domains in Health Care

At our institution, we have used dozens of frameworks to implement hundreds of applications representing the three CDS domains outlined above. Herein, we present the successes and challenges we observed with the application of these frameworks.

### Population-Level CDS

Our medical center is expanding its population health care management services to evolve with the growing focus of payers on management of high-risk patient groups and on performance metrics regarding integrated care delivery. This is being accomplished in part by the creation of EMR patient registries for chronic diseases such as diabetes, chronic obstructive pulmonary disease (COPD), and heart failure. Using regional health information exchanges, the statewide all-payer claims database, and our institutional clinical data warehouse, these registries integrate comprehensive data regarding clinical encounters, management changes, medication use, and outcomes both within and outside of our health care system for patients in each registry. We are developing CDS alerts based on these diverse data and on patient assessments and previous intervention outcomes to trigger tasks and education goals for the patient. For example, patients in a diabetes registry may have visit documentation and lab results stored in the EMR. If a patient’s routine assessments include elevated hemoglobin A1C and poor familiarity with home monitoring equipment, the care manager receives a notification recommending goal-setting with the patient (eg, for home monitoring and stabilizing the patient’s hemoglobin A1C) and adding tasks (eg, an order for a blood glucose monitor, future lab tests, or patient education). Although the role of CDS in the population health domain is growing, challenges remain due to the lack of established standards and provider workflows for care management, resulting in inconsistent delivery of notifications to the right people or at the right time and in the right format.

### Encounter-Level CDS

**Figure 1:**
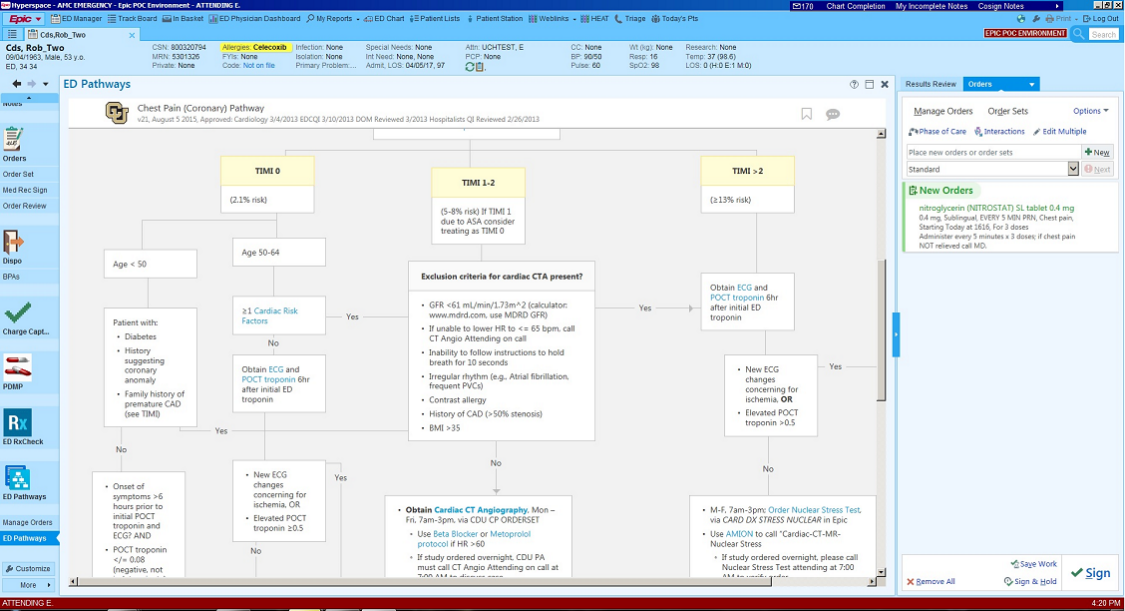
Example of an encounter-level care pathway clinical decision support application using synthetic patient data.

Variability in management of common diagnoses such as pneumonia, heart failure, and COPD exacerbation may lead to poor outcomes. Conversely, improved standardization of care can improve outcomes [21,22]. Care pathways are one method of encouraging treatment standardization to improve patient outcomes while permitting flexibility within specific patient presentations [23]. They are often presented as workflows, prompting providers to adhere to recommended care practices. Historically, these pathways have been represented as static flowcharts that the provider can follow in a stepwise fashion, often as a printed diagram or as a digital picture. However, this method is inefficient and the flowcharts are often overlooked, as the decision aid is not made directly available in a provider’s workflow. At our institution, we have embedded evidence-based care pathways within the EMR using web-based content in the clinical workflow for evaluation and treatment of patients presenting with common chief concerns such as chest pain or headache. The interface provides an interactive decision tree–like diagram similar to historic representations of care pathways to facilitate standardized patient management. However, the visual tool is implemented using a combination of native EMR functionality and third-party vendor technology, which allows the provider to place orders, perform calculations, and await results. CDS care pathways have been implemented most extensively in our ED. For example, the EMR will present a chest pain care pathway to the provider within the EMR record of a patient reporting a chief concern of chest pain (Figure 1). The provider navigates through the diagram workup steps and initial treatment (eg, aspirin, beta-blocker, nitroglycerin), and an interactive calculator is presented for the Thrombolysis In Myocardial Infarction score, which is a simple risk-stratification algorithm for non–ST-segment elevation myocardial infarction and unstable angina [24].

The care pathway links to an order placement queue for actions such as medications or additional tests, which is provided inline within the EMR. Importantly, orders can be placed directly from the flow diagram rather than requiring the provider to switch back and forth between applications or refer back to a printed diagram when taking a clinical action. Instead, the decision support is provided directly within the provider’s workflow. Later in the encounter, the provider can return to the pathway at any given step (e.g., when new test results become available). By embedding order recommendations based on risk stratification within the care pathway, adherence to institutional care standards becomes the most efficient clinical workflow for the provider while preserving the ability to take alternative actions that may be warranted by the clinical scenario. Seamless integration of CDS technology within the EMR workflow provides a straightforward way to standardize care that promotes adherence to guideline-based therapy.

Important challenges in care pathway development include limitations of native EMR functionality in terms of dynamic data collection (eg, provider-entered data), visualization technology, and ability to translate clinical decisions into action. At our institution, implementation of these care pathways was made possible only by partnering with a third-party CDS vendor who developed the EMR interface as well as the data capture, analysis, and visualization technologies to achieve seamless clinical workflow integration. Based on our experiences with third-party technology for care pathways, we anticipate that the ability of a system to integrate with content and technology vendors will greatly expand options for innovative use of CDS.

Well-described predictive models can provide a specific assessment of a patient’s risk using a real-time EMR. Implementing CDS based on risk scores can support evaluation and intervention using guideline-directed therapy. EMRs now include some risk models as part of their native functionality, such as the Length of stay, Acuity of admission, Comorbidities, Emergency department (LACE+) model or Early Warning Scores (EWS) [25-29]. Simple applications can alert case managers or nursing supervisors, respectively, when a patient’s risk score exceeds a specific threshold, prompting further patient evaluation. For example, we use EWS on medical-surgical wards for early identification of patient deterioration. Score-driven CDS is calculated and presented to the patient’s nurse manager, who generates a rapid response alert to evaluate the patient. Recent advances in EWS CDS systems have enabled real-time collection of patient vital signs and more diverse clinical data, more frequent calculation of EWS, and use of more complex models that predict clinical deterioration, allowing interventions to be initiated earlier to prevent or minimize adverse outcomes.

Important considerations include the timing, amount, and reliability of the data used for these scores. Data collection can be achieved directly from the EMR via third-party collection devices imported to the EMR or by manual entry, and significant challenges exist in extracting accurate data from the EMR or when providers are required to enter additional data to support complex predictive models [30]. New tools are in development to address these challenges and improve the quality of data used in these risk scores, coordinate various data sources, optimize entry and extraction of data with the EMR, and support implementation of larger libraries of complex models.

As with all CDS, a significant challenge in generating specific application requirements is optimization of CDS-workflow integration. Application requirements can be highly specific to disease processes, clinical venues, provider groups, or institutions, all of which must be considered. Furthermore, the same data (eg, risk assessment) may need to be delivered in a variety of ways depending on the care venue and providers. For the same CDS result, in some cases, an interruptive alert during a clinic visit may be preferred for physicians, a work list element may be preferred for care mangers, and an educational email may be preferred for a patient. The variable requirements of different CDS applications require a wide range of technical functionality. We addressed this by developing an array of CDS tools, including the EMR, third-party software, and custom applications built in-house in collaboration with a clinical champion.

### Precision-Level CDS

Many existing CDS applications have been directed at providers based on coarse classifications (eg, presence of diabetes or hospitalization for acute myocardial infarction). With increasing focus on shared decision-making, emerging CDS tools are directed at providing individualized assessments and predictions to facilitate complex discussions between patients and providers. Dependence on many varied data inputs can discourage clinicians from using such models on a practical basis despite their superior predictive ability. Novel data streams such as genomic analyses and fitness trackers, or the variety of data requirements for highly accurate predictive algorithms, can eclipse human capacity to comprehensively process data. CDS analytics are therefore essential to synthesize the amount, breadth, and complexity of data necessary for precision medicine, such as in the cases of genomic medicine, data from wearable devices, and complex patient-reported outcome instruments.

As the least well-developed of the three domains, precision medicine CDS faces many challenges. There is relatively little knowledge or understanding of how to implement and use applications requiring myriad data points to generate highly patient-specific management plans, and developing this knowledge is outside the capacity of most health care organizations. Precision medicine strategies may need to both educate and support providers in decision-making where the foundational knowledge to create the CDS is not widespread. Finally, optimal methods of patient engagement (eg, format and mode of delivery) using this information are not yet well understood.

At our institution, we have approached these complex challenges by developing tools such as an interactive CDS application in collaboration with members of the Surgical Outcomes and Applied Research Program within the Department of Surgery. This application is based on the published Surgical Risk Preoperative Assessment System (SURPAS), which is a set of risk predictive algorithms developed from the American College of Surgeons National Surgical Quality Improvement Program data to predict 11 adverse postoperative outcomes using 8 preoperatively available predictor variables [31].

**Figure 2:**
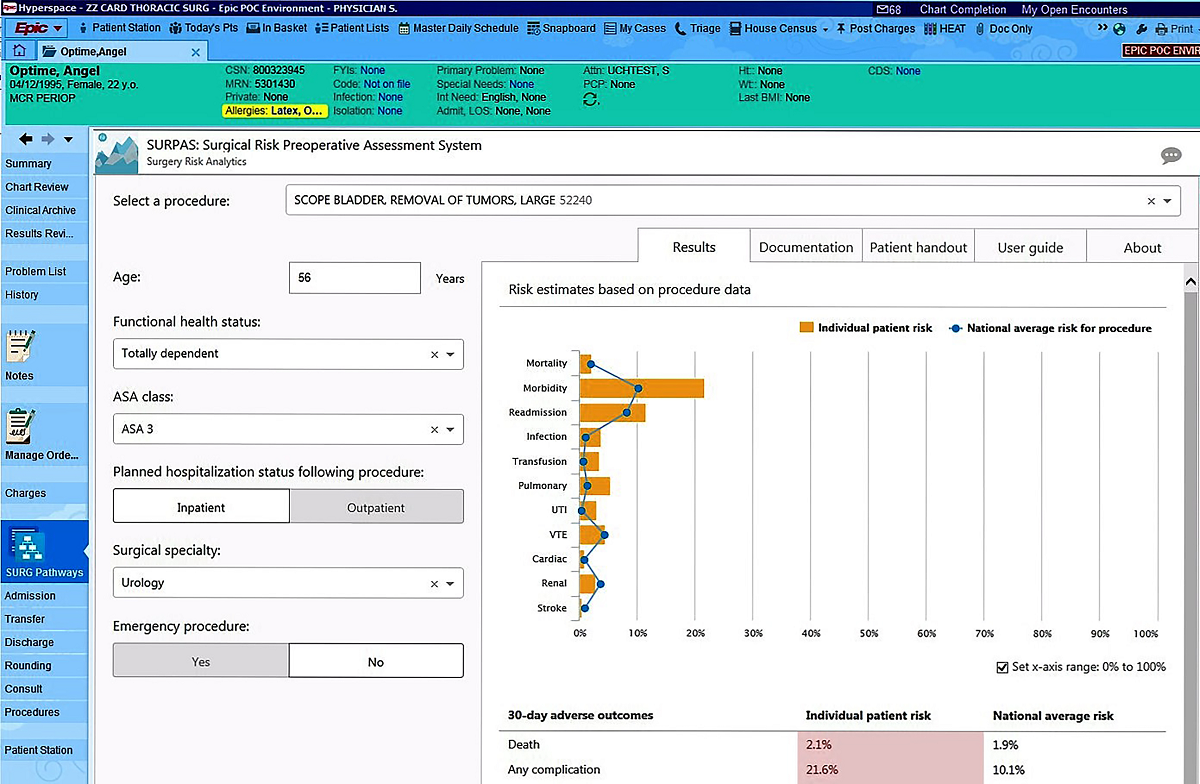
Screenshot of the Surgical Risk Preoperative Assessment System, a personalized risk assessment clinical decision support application used to guide postoperative care, with synthetic patient data.

The SURPAS intervention allows individualized inputs into the model that provide a precise and personalized risk assessment instead of a categorized level of risk. Using this method ensures precision medicine CDS in which the model will not make the same recommendations for different patients. Within the EHR, SURPAS automatically combines previously existing EHR data for each patient with provider-entered patient data to calculate the patient’s procedure-specific likelihood of 11 different surgical complications. Then, the patient- and procedure-specific risk assessments are compared to national averages ranging from renal injury to 30-day mortality. During the preoperative office visit, patients are presented with their individualized risks of postoperative adverse outcomes as an infographic education tool, which streamlines the risk discussion and consent process, encourages patient engagement, and alerts providers to individual patient risk profiles that may guide preparations for postoperative care (Figure 2). Finally, risk estimates can be imported directly into clinic notes to document the basis for patient-centered care decisions.

**Figure 3:**
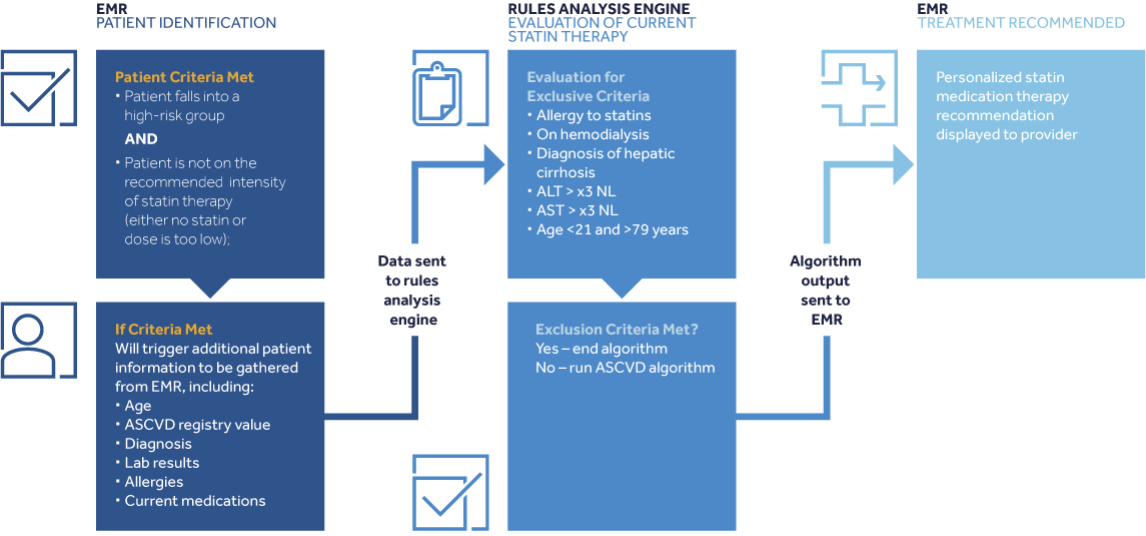
An atherosclerotic cardiovascular disease risk scoring algorithm to classify patients according to their risk group. ALT: alanine aminotransferase; ASCVD: atherosclerotic cardiovascular disease; EMR: electronic medical record; NL: normal.

Precision-level CDS can also provide recommendations in the context of both complex risk assessment and a patient’s current management. For example, our institution leverages EMR data to generate alerts based on risk modeling to identify patients for initiation or modification of cholesterol management protocols. In 2013, the Adult Treatment Panel IV on cholesterol management updated practice guidelines for patients with prevention of atherosclerotic cardiovascular disease (ASCVD). The guideline now provides recommendations for determining the appropriate intensity of statin therapy based on four recommended risk groups, one of which is defined by a complex ASCVD risk model [32]. We have built a multimodal CDS application that uses extracted data, including the ASCVD risk score, to classify patients according to four specified risk groups to determine the recommended intensity of statin therapy, if any. The algorithm then evaluates the presence and intensity of ongoing statin therapy to generate a recommendation to the provider only if a change in statin therapy is indicated. The results and recommendations are visualized as alerts presented to the provider via the EMR during the encounter to facilitate guideline-based care (Figure 3).

## Discussion

### CDS Implementation Across All Three Domains

**Figure 4:**
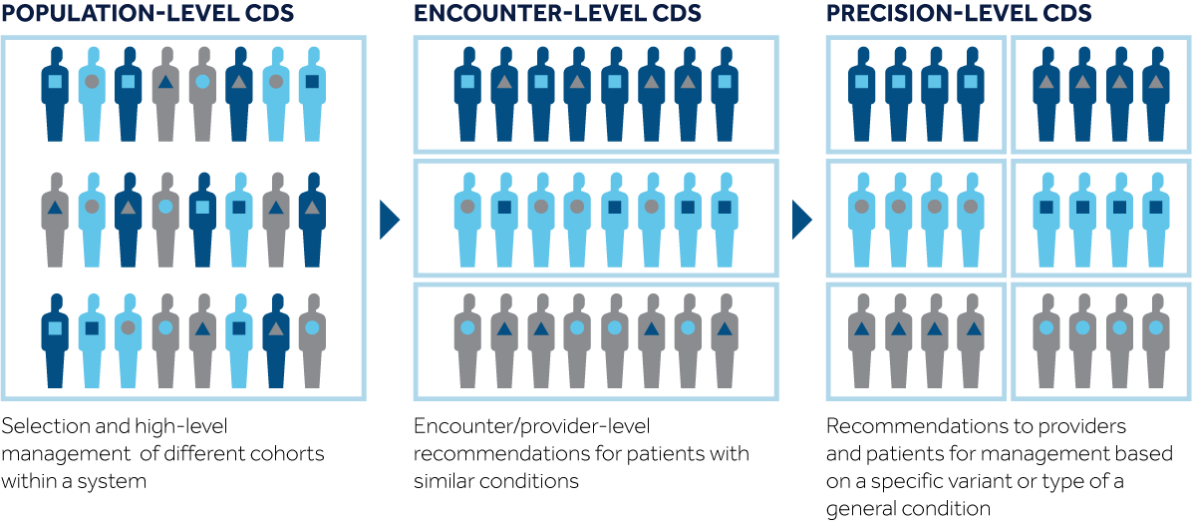
Overview of the integration and relationship of CDS across three domains of health care. CDS: clinical decision support.

Care delivery across all three clinical care domains using integrated CDS applications has the potential to improve efficiency and quality for individual patients; however, significant planning and development effort is required to ensure that applications align with several types of end-user workflows. The three different levels of CDS have distinct, valuable roles with specific requirements and functionality (Table 1). All 3 levels should be used in concert when optimizing and coordinating a patient’s care. Figure 4 presents a construct demonstrating the relationship of these care domains. Although the diagram flows from population to encounter to precision domains, in practice, workflows may practically or temporally move across the domains in any order, depending on the algorithm and the condition in question. The person requesting an application as well as the intended end users should ideally be engaged in the development process to optimize integration into the providers’ workflow and to maximize provider uptake. Historically, the available technology has determined the types of CDS applications that can be requested; however, as the CDS capabilities of an institution grow, clinical use cases increasingly determine the selection of technical approaches from the organization’s “toolbox.”

**Table 1:** Salient features of each domain according to each level of CDS.

Feature	CDS^a^ level		
	Population	Encounter	Precision
Alert timing	Asynchronous	Synchronous	Both
Data timing	Cumulative	At time of encounter	Preemptive
Basis	Evidence-based	Evidence-based	Individualized
Strategies	Dashboards, iterative risk scores, work lists	Interruptive alerts, risk scores, care pathways, passive alerts	Either population- or encounter-based
Clinical examples	Diabetic foot exam, HIV drug efficacy, annual cholesterol	Heart failure guidelines, QT-prolonging drugs, performance metrics, deterioration index	Telemonitoring and mobile health, *CYP2D6*/opiate metabolism, polygenic risk scores, BRCA cancer screening

^a^CDS: clinical decision support.

As an example, our institution is developing an end-to-end, multi-tiered precision medicine framework that will use complex predictive models that integrate clinical data with a patient’s genetic profile (ie, a single gene or a few genes) or genomic profile (ie, many or all genes) to assist in clinical decision-making. First, patients undergo genome-wide single nucleotide polymorphism analysis to produce clinically actionable genetic data, such as genes that increase disease risk or impact drug effectiveness. These data are then returned to the patient’s individual record in the EMR, which may occur long before the genes become clinically relevant (eg, when a clinician attempts to prescribe a specific medication whose effectiveness could be influenced by the patient’s genotype). CDS applications will use these data to support population care management, encounter-based tools, integrated third-party data collection methods, and genetic and genomic data, producing result displays that are customized separately for patients and providers. At a population level, high-risk patients will be identified with the help of select genetic data that are relevant for assessing the patients’ risk of developing certain diseases. Care pathways relevant to these high-risk populations may recommend increased intensity of disease screening or review of possible preventive interventions. Assigned care managers will coordinate long-term care management for those patients; this may include appropriate proactive care plans such as lifestyle modifications, genetic counseling, family education, or frequent diagnostic screening tests. At the encounter level, CDS will alert providers when ordering a medication affected by a patient’s genetics by providing real-time feedback to the clinician using evolving evidence regarding relevant pharmacogenetic markers. Patient education and engagement will be enhanced by combining genetic information with the results of risk modeling algorithms. This information will be shared with patients along with educational resources and genetic counseling as appropriate, facilitating collaborative decision-making between the patient and provider. This framework has required contributions from a wide array of experts, including basic genomic scientists, the pathology laboratory and clinical laboratory systems, clinical informaticians, pharmacists, EMR technical staff, patient representatives, hospital legal representation, and ethics committees. We have also greatly increased the technical capacity of our EMR to return the genetic data in a format that can be used by all three levels of CDS.

To illustrate how CDS can impact an individual patient through the three care domains, we present the trajectory of LK, a hypothetical patient with COPD, at different stages of care management in Table 2. Although this specific example is theoretical, we have developed similar applications at our institution at each level of care for different disease domains as described above. We use the example of LK to cohesively illustrate how all these applications could be integrated to support a comprehensive, holistic approach to the care of her chronic disease. Management of LK’s COPD used population-level CDS via the registry (COPD), management protocols (annual spirometry and symptoms), and alerts regarding both planned (care provider) and unplanned (ED or inpatient) patient encounters to coordinate care (eg, home health evaluation). The encounter-based CDS helped providers choose and implement the correct guideline-based medications for different scenarios (corticosteroid for worsening COPD, cefepime when hospitalized for COPD, hydrocodone vs codeine based on CYP2D6 genotype, influenza vaccine on hospital discharge). Precision-level CDS helped refine management based on LK’s specific characteristics including her genetics (selection of cefepime based on age and forced expiratory volume in first second of expiration [FEV1] selection of hydrocodone, and use of capnography monitoring based on CYP2D6). In this case, each CDS domain provided a level of integrated care coordination to manage LK’s COPD and contributed significantly to LK’s management and improved quality of care.

**Table 2:** Integration of CDS across the population, encounter, and precision care domains of LK, a hypothetical 68-year-old female patient with COPD.

Care management action	Associated CDS^a^ level
LK is assigned a care management team (disease registry) that monitors her clinical status using annual office spirometry.	Population
After 3 years, longitudinal analytics alert LK’s care managers that her spirometry is declining and her symptoms are increasing.	Population
Based on this trend, the team schedules an appointment with her health care provider. The provider considers starting a long-acting beta-agonist alone, but when he tries to order one, he is prompted to start an inhaled corticosteroid in accordance with present guidelines.	Encounter
After 6 months, LK has a severe COPD^b^ exacerbation. She contacts her care team through an EMR^c^, and they advise her to go to the emergency department.	Population
When LK is admitted to the hospital, the EMR recommends intravenous cefepime because she meets the criteria for complicated COPD based on her age of older than 65 years and a recent spirometry FEV_1_^d^ measurement of less than 50% predicted. During her hospitalization, LK develops a rib fracture from coughing and has severe pain. A genomic analysis performed two years earlier as part of the institution’s precision medicine program determined that she had multiple copies of the *CYP2D6* gene, indicating an increased likelihood of excessive sedation from codeine-containing cough syrups due to rapid conversion into morphine.	Encounter and precision
The hospitalist is alerted to her pharmacogenetic status and prescribes hydrocodone instead of codeine for management of pain and cough, and capnography monitoring is used to monitor for respiratory depression or failure.	Encounter and precision
LK is ready for discharge after 5 days. Based on her known COPD and hospitalization, the EMR recommends an influenza vaccine prior to discharge.	Population and encounter
The discharging team arranges follow-up with LK’s primary care provider. Her chronic care managers receive an alert that she is being discharged and contact her three days later. Through a video call, they learn that she is having trouble with daily activities due to deconditioning and the rib fracture. A home health evaluation is arranged, and physical therapy and home health nursing are prescribed. LK improves over the next 2 weeks and returns to her baseline surveillance schedule.	Population

^a^CDS: clinical decision support.

^b^COPD: chronic obstructive pulmonary disease.

^c^EMR: electronic medical record.

^d^FEV_1_: forced expiratory volume in first second of expiration.

### Conclusions

CDS is challenging to design and implement; however, significant progress has been made, with improvements in timely and workflow-specific management recommendations, EMRs, and resources created by third-party vendors. Conceptualizing CDS tools in the context of linked population-, encounter-, and precision-level health care affords an opportunity to integrate complex algorithms at each level into a unified mechanism for improving care across all levels of patient management.

## Abbreviations

ASCVD: atherosclerotic cardiovascular disease
CDS: clinical decision support
COPD: chronic obstructive pulmonary disease
ED: emergency department
EMR: electronic medical record
EWS: Early Warning Scores
FEV1: forced expiratory volume in first second of expiration
LACE+: Length of stay, Acuity of admission, Comorbidities, Emergency department
SURPAS: Surgical Risk Preoperative Assessment System
